# Efficacy of Stochastic Vestibular Stimulation to Improve Locomotor Performance During Adaptation to Visuomotor and Somatosensory Distortion

**DOI:** 10.3389/fphys.2018.00301

**Published:** 2018-03-29

**Authors:** David R. Temple, Yiri E. De Dios, Charles S. Layne, Jacob J. Bloomberg, Ajitkumar P. Mulavara

**Affiliations:** ^1^Department of Health and Human Performance, University of Houston, Houston, TX, United States; ^2^Center for Neuromotor and Biomechanics Research, University of Houston, Houston, TX, United States; ^3^KBRwyle, Houston, TX, United States; ^4^Center for Neuro-Engineering and Cognitive Science, University of Houston, Houston, TX, United States; ^5^Johnson Space Center, National Aeronautics and Space Administration, Houston, TX, United States

**Keywords:** stochastic resonance, vestibular, locomotion, somatosensory, vision

## Abstract

Astronauts exposed to microgravity face sensorimotor challenges affecting balance control when readapting to Earth's gravity upon return from spaceflight. Small amounts of electrical noise applied to the vestibular system have been shown to improve balance control during standing and walking under discordant sensory conditions in healthy subjects, likely by enhancing information transfer through the phenomenon of stochastic resonance. The purpose of this study was to test the hypothesis that imperceptible levels of stochastic vestibular stimulation (SVS) could improve short-term adaptation to a locomotor task in a novel sensory discordant environment. Healthy subjects (14 males, 10 females, age = 28.7 ± 5.3 years, height = 167.2 ± 9.6 cm, weight = 71.0 ± 12.8 kg) were tested for perceptual thresholds to sinusoidal currents applied across the mastoids. Subjects were then randomly and blindly assigned to an SVS group receiving a 0–30 Hz Gaussian white noise electrical stimulus at 50% of their perceptual threshold (stim) or a control group receiving zero stimulation during Functional Mobility Tests (FMTs), nine trials of which were done under conditions of visual discordance (wearing up/down vision reversing goggles). Time to complete the course (TCC) was used to test the effect of SVS between the two groups across the trials. Adaptation rates from the normalized TCCs were also compared utilizing exponent values of power fit trendline equations. A one-tailed independent-samples *t*-test indicated these adaptation rates were significantly faster in the stim group (*n* = 12) than the control (*n* = 12) group [*t*_(16.18)_ = 2.00, *p* = 0.031]. When a secondary analysis was performed comparing “responders” (subjects who showed faster adaptation rates) of the stim (*n* = 7) group to the control group (*n* = 12), independent-samples *t*-tests revealed significantly faster trial times for the last five trials with goggles in the stim group “responders” than the controls. The data suggests that SVS may be capable of improving short-term adaptation to a locomotion task done under sensory discordance in a group of responsive subjects.

## Introduction

In astronauts, prolonged exposure to microgravity induces an adaptation to that environment resulting in reinterpretation of visual, vestibular, and somatosensory inputs (Paloski et al., 1992, 1994; Reschke et al., 1994; Bloomberg et al., 2015). Upon return to a gravitational environment, postural control and balance can be severely compromised until the central nervous system (CNS) readapts to correctly process sensory information from a terrestrial environment (Paloski et al., 1992). Walking ability may take as long as 15 days to be fully restored upon returning to Earth (Mulavara et al., 2010). There are two periods in which adaptation is commonly observed in individuals: (1) rapid, within-session trial by trial improvements when completing a task multiple times, and (2) slower evolving incremental performance gain often observed over multiple practice sessions (Mulavara et al., 2010). The processes by which these rapid and slower adaptation curves occur are often referred to as strategic control and adaptive realignment respectively (Redding and Wallace, 2002; Richards et al., 2007; Mulavara et al., 2010). These two processes are considered interdependent (Redding and Wallace, 2002; Richards et al., 2007), and longer-term locomotor adaptive recovery has been shown to be associated with short-term strategic capabilities of astronauts readapting from long-duration spaceflight. Specifically, those astronauts demonstrating faster short-term (strategic) adaptation rates 1 day after their return also show faster overall recovery (Mulavara et al., 2010). If effective countermeasures can be implemented that develop faster short-term improvements in balance and locomotor skills, populations such as astronauts might be able to utilize strategic responses to speed recovery from adaptation to prolonged microgravity exposure after gravitational transitions. One proposed countermeasure, which has been shown to benefit sensory system capabilities and associated performance improvements, is through the addition of small amounts of noise via a phenomenon known as stochastic resonance (SR).

Despite the commonly held belief that noise is a hindrance to signal detection, in more recent years SR has been suggested as a means by which recognition of weak sensory input signals may be enhanced by the addition of an appropriate amount of noise (Moss et al., 1994, 2004; Wiesenfeld and Moss, 1995; McDonnell and Abbott, 2009; Aihara et al., 2010). SR occurs in non-linear systems when addition of noise results in improved signal transmission or detection (Collins et al., 2003; Moss et al., 2004; McDonnell and Abbott, 2009). Recently this phenomenon has been explored with the idea of improving physiological systems through optimizing neuronal noise (McDonnell and Abbott, 2009). Some studies have noted changes in autonomic responses attributed to SR, such as enhanced heart rate and muscle sympathetic nerve activity under conditions of hypovolemic stress, which likely resulted from improved baroreceptor signaling by adding noise directly via carotid sinus baroreceptors (Hidaka et al., 2000, 2001; Yamamoto et al., 2002). Additionally, SR using imperceptible stochastic electrical stimulation of the vestibular system, applied to normal subjects, has been shown to improve the degree of association between the weak input periodic signals introduced via venous blood pressure receptors and the heart rate responses (Soma et al., 2003). When the stochastic current is applied to the vestibular system over 24 h, SR improves the long-term heart rate dynamics and motor responsiveness as indicated by daytime trunk activity measurements in patients with multisystem atrophy, Parkinson's disease, or both, including patients who were unresponsive to standard levodopa therapy (Yamamoto et al., 2005). If detection of weak sensory signals can be improved through SR, then the sensorimotor coordination which accounts for the maintenance of equilibrium also stands to benefit. One proposed hypothesis is that noise generates small changes in receptor transmembrane characteristics allowing the detection of a weak stimulus (Gravelle et al., 2002; Collins et al., 2003; Mulavara et al., 2011). There is evidence that all three sensory systems responsible for balance (i.e., vision, somatosensation, and vestibular) are capable of improved detection of weak sensory signals through SR, thus helping improve performance of balance control (Collins et al., 2003; Priplata et al., 2003, 2006; Sasaki et al., 2006, 2008; Aihara et al., 2008; Pal et al., 2009; Mulavara et al., 2011; Goel et al., 2015). Numerous studies have shown the signaling capacity of somatosensory afferents to be enhanced with the addition of noise stimuli delivered just at or below that of perceptual thresholds (Collins et al., 1996a,b; Dhruv et al., 2002; Liu et al., 2002; Khaodhiar et al., 2003). There is evidence that enhancing signal detection of cutaneous afferents, with the addition of subthreshold noise delivered via a mechanical stimulus such as vibration to the soles of the feet can improve balance (Priplata et al., 2002, 2003, 2006) and locomotion performance (Galica et al., 2009; Stephen et al., 2012). A few studies have noted improvements in balance control during standing and walking when using imperceptible amounts of stochastic electrical current delivered through the vestibular system, which are likely occurring through means of SR both in healthy controls and in patients with a variety of neurological disorders (Pal et al., 2009; Mulavara et al., 2011, 2015; Goel et al., 2015; Samoudi et al., 2015; Wuehr et al., 2016a,b). Stochastic vestibular stimulation (SVS) was found to decrease anterior-posterior (A/P) sway in Parkinson's Disease patients when a small stochastic current (max amplitude of 0.1 mA) was delivered across the vestibular end organs by placing two cathodes on the mastoid processes and an anode over the C7 vertebra. The improvement was very small (4.5%), although a significant enhancement in a normal population was not noted (Pal et al., 2009). More recently, balance performance with SVS delivered in a mediolateral (M/L) fashion (binaural bipolar vestibular stimulation with electrodes placed directly over the mastoid processes) has been shown to improve balance while standing on a foam compliant surface (Mulavara et al., 2011; Goel et al., 2015). Likewise, using similar amplitudes of M/L SVS during locomotion improved walking stability in patients with bilateral vestibulopathy (Wuehr et al., 2016a) as well as normal healthy subjects (Mulavara et al., 2015; Wuehr et al., 2016b).

To determine if SVS can enhance short-term strategic adaptation in a locomotion task, we used subthreshold bipolar binaural SVS in a novel visual and somatosensory discordant environment. We hypothesized that subthreshold electrical vestibular stimulation with white Gaussian distributed noise SVS would significantly improve locomotor adaptation to a novel sensory discordant environment, compared to controls receiving zero vestibular stimulation.

## Methods

### Participants

A sample of 27 healthy individuals (15 males, 12 females, age = 28.9 ± 5.0 years, height = 167.7 ± 9.2 cm, weight = 72.4 ± 15.0 kg), with no known musculoskeletal or neurological deficits were recruited from the Department of Health and Human Performance at the University of Houston. Written informed consent was obtained from each subject prior to the start of the experimental procedures. Approval to conduct this study was granted by the Committees for the Protection of Human Subjects at the University of Houston, which conforms to the Declaration of Helsinki.

### Procedures

All subjects were required to fill out a physical activity readiness questionnaire (PAR-Q) prior to the study session. Individuals were excluded if the PAR-Q indicated they had any known neurological dysfunction, recent bouts of vertigo, bone or joint issues, prior bad experience with Galvanic vestibular stimulation, poor vision, were pregnant, diabetic, epileptic, had balance or gait problems, or had any major surgeries recently that might impact their balance or locomotion. Height, weight, shoulder height, and shoulder width were measured on all the subjects just prior to preparing them for the electrode placement. Although not a prerequisite for the study, all subjects were right-hand dominant.

#### Electrode placement

All subjects sat in a chair while the skin over the mastoid processes were cleaned and prepared for electrode placement. Two 5 × 10 cm electrodes (Axelgaard Manufacturing, Fallbrook, CA, USA) coated with a thin layer of Signa Gel® (Parker Laboratories Corp., Fairfield, NJ, USA) were centered over the two mastoid processes and two soft foam pads were then placed over the electrodes and secured in place by a head strap. Impedance between the electrodes was always confirmed to be less than 1 kΩ.

#### Thresholding task

After electrode placement, a thresholding task designed to identify the level of electrical vestibular stimulation at which subjects could discern head motion induced by the stimulation was conducted, using methods described in previous papers (Goel et al., 2015; Mulavara et al., 2015). Subjects sat on a stool without a backrest, with their feet on the footrest, and held a gamepad (Logitech Gamepad F310, Lausanne, Switzerland). Utilizing the gamepad, they indicated their ability to perceive a sinusoidal bipolar stimulus current (which produces a side-to-side head motion sensation) applied between the electrodes. The exact instruction given to each subject was: “Use your dominant hand to push a joystick depending upon the direction of the motion sensation; make sure to do it as long as you feel the sensation”. A 1 Hz sinusoidal electrical stimulation signal was chosen for motion threshold determination in this study. In general, the stimulus profile consisted of 15 s periods with the 1 Hz sinusoidal stimulation signals, interspersed with 20–25 s periods of no stimulation. The different current peak amplitudes used were 100, 200, 300, 400, 500, 600, 700, 900, 1100, 1300, 1500 μA. The order of the stimulation levels, within the profile was randomized for all subjects. Total duration for this task was 463 s. For the joystick data, percentage time of “perceived motion reported by the subject” for each stimulation and baseline periods was calculated. Joystick movement was interpreted as “perceived motion reported by the subject,” when its output amplitude exceeded 0.05 V (full-scale movement recorded in 0–5 V range). The percentage time at each stimulation and baseline level for perceptual and body motion detection was normalized with respect to the largest value across all levels of stimulation. A binomial distribution function was fit to the data with a generalized linear model and a logit link function, which is very common in psychophysical studies (Treutwein, 1995). Threshold was defined as the amplitude of stimulation at the point of subjective equality, at which there is a 50% chance of motion detection.

Upon finishing the thresholding task and calculating a subject's perceptual threshold, subjects were then randomly assigned to one of two groups: those receiving maximum amplitude SVS at 50% of their calculated perceptual threshold (stim), and those receiving zero stimulation (control). Post analysis two-tailed independent-samples *t*-tests confirmed that the two groups did not differ significantly in anthropometric measures (height, weight, shoulder height, and shoulder width), perceptual threshold levels, or baseline times for the Functional Mobility Test (FMT) without goggles. Those who received zero stimulation were given zero amplitude of current delivered to their electrodes, while those who received the SVS (stim) were given a zero mean, Gaussian distributed white noise electrical signal in a wideband 0–30 Hz frequency range during all trials of the locomotion task. In both groups, the device was switched on just prior to subject beginning the course. Root mean square (RMS) of the signals was checked to be [(26 μA RMS/100 μA peak) ± 5%]. This type of wide-band vestibular noise has been used in prior studies and found to benefit balance metrics in both static stance (Mulavara et al., 2011; Reschke et al., 2014; Goel et al., 2015) and dynamic locomotor control (Mulavara et al., 2015). We have reasoned with supporting evidence from other studies (Dakin et al., 2007; Songer and Eatock, 2013) that the wideband range of 0–30 Hz can improve postural control performance in standing and walking tasks by stimulating vestibular hair cells (VHCs) that affect posture and evoke vestibulo-myogenic response in the lower limbs (Mulavara et al., 2011, 2015; Goel et al., 2015).

#### Locomotion task/functional mobility test (FMT)

All subjects performed 12 trials of a locomotor assessment consisting of a slightly modified version of the FMT, which has been previously used to assess locomotor capabilities of astronauts returning from long-duration spaceflight (Mulavara et al., 2010; Cohen et al., 2012), bedrest subjects (Reschke et al., 2009), and healthy individuals alike (Moore et al., 2006; Mulavara et al., 2009). All subjects wore the portable current stimulator and electrodes during 12 FMT trials. Neither group reported any sensations of electrical stimulus throughout the FMT trials. Although the stim group did receive SVS during their trials, it was below their perceptual threshold and not perceptible. Thus, subjects were blinded to which group they were in. The 12 FMT trials consisted of three baseline (*B*_1_ – *B*_3_) trials without vision distortion and nine goggle (*G*_1_ – *G*_9_) trials performed with subjects wearing vision reversing prism (up/down) goggles in order to provide visual discordance (vision is distorted by the goggles). The entire FMT course was performed on 10-cm thick foam to make somatosensory/proprioceptive input unreliable to the subjects and provide a greater postural challenge. In addition to providing a sensory discordance, the foam also served as an added safety benefit. If any subjects were to fall, it created a soft landing surface to prevent injury. Thus, in trials with visual discordance, the most reliable feedback system supporting balance control was the vestibular system. The sensory discordant conditions of the FMT provided an ideal amount of challenge in most subjects, allowing us to observe adaptation curves in our primary metric, time to complete the course TCC in seconds.

**Figure 1:**
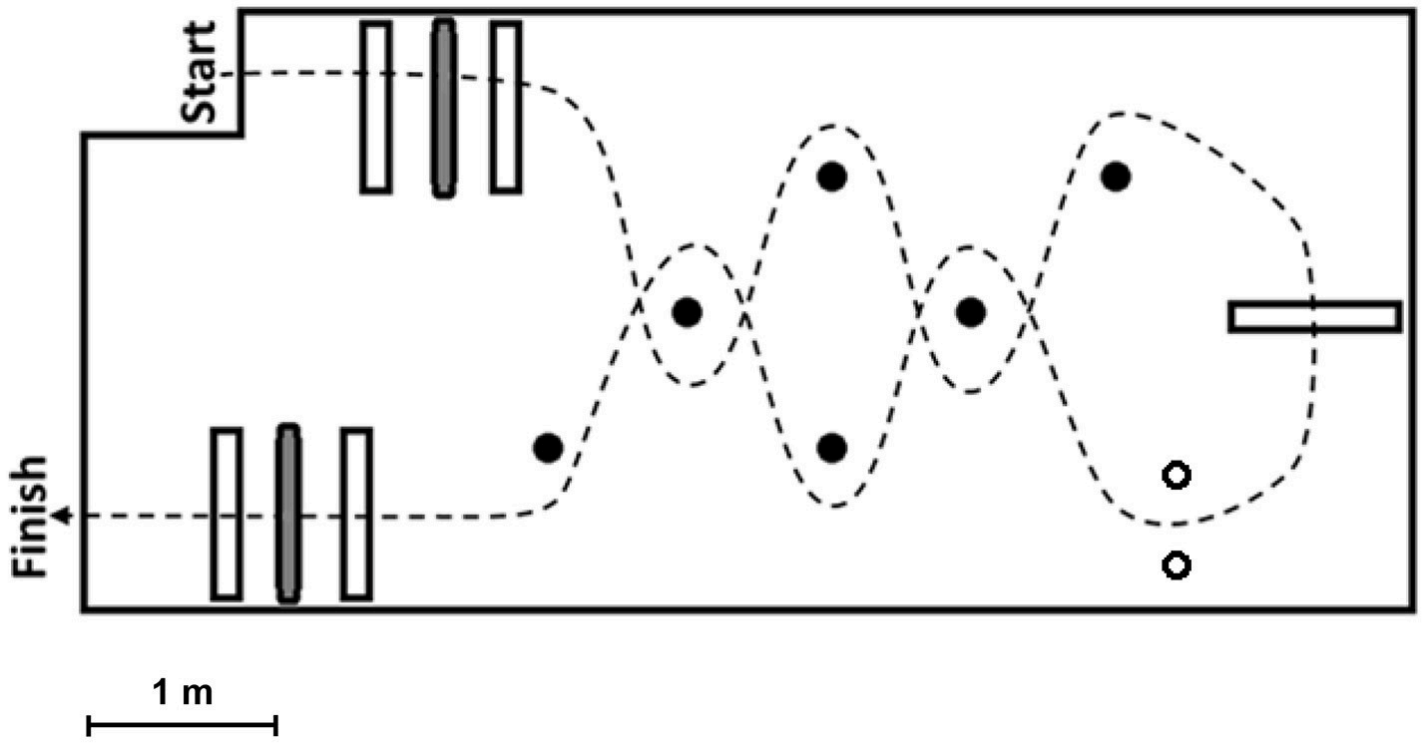
Depicts the setup for the Functional Mobility Test (FMT). White bars indicate obstacles the subjects had to step over, while gray bars represent horizontal obstacles hung from the ceiling which required subjects to duck under them. Black circles show vertical poles the subjects had to navigate around during the “slalom” sections, and the two white circles represent the poles hung vertically from the ceiling that comprised the narrow “gate” subjects attempted to squeeze through. The course path subjects were required to take is indicated by the dashed line.

Subjects wore socks and were instructed to navigate the FMT course as quickly as they could without running or touching any obstacles (Figure 1). Before each FMT trial, subjects were walked to a starting line approximately six inches from the foam surface. Both subjects' feet were moved up to a position just touching this start line prior to beginning. Timing of a trial began on a subject's first movement. Subjects then stepped onto the foam and toward the first “portal” obstacle, which consisted of two successive 31-cm high Styrofoam blocks placed on the foam surface with a horizontal bar hung from the ceiling between the two blocks at a height adjusted to that of the subject's shoulders. The “portal” required subjects to bend at the waist or lower themselves to avoid hitting the bar and balance on a single foot on the compliant surface while they stepped over the Styrofoam barriers. Next a “slalom” section consisted of four foam poles placed vertically from the floor, which made subjects change head directions and challenged their spatial awareness as they navigated around the poles. A larger 46-cm high Styrofoam block was then placed after the first “slalom” section, and it again required subjects to balance with one foot on the compliant surface as they stepped over. After stepping over, subjects then turned and went through a narrow “gate” which consisted of two foam poles hung vertically from the ceiling at a distance set to the subject's shoulder width. Subjects often elected to go through the “gate” sidewise in an attempt to not touch the poles. Once making it through the “gate,” subjects then came back through a second “slalom” and “portal” section. Timing of the subjects stopped once both feet touched down on the hard surface located just after the last “portal” section.

After each trial of the FMT was performed, subjects were asked to rate on a scale of 1–5 (with one indicating none and five indicating a great deal) sensations of electrode irritation, nausea, and their degree of difficulty balancing. Additionally, after all FMT trials were performed, subjects were asked to indicate again on the same scale of 1–5 if they had any sensations of pain, tingling, itching, burning, vertigo, fatigue, nervousness, difficulty concentrating, changes in headache perception, general unpleasantness, or visual sensations throughout the FMT trials. Subjects had also been asked to rate these 11 sensations immediately after threshold testing in order to gauge if subjects were experiencing any adverse effects from the vestibular stimulation.

### Data analysis

There were no significant differences in TCC between the stim and control group for any of the three baseline trials (*B*_1_ – *B*_3_), and they were therefore not included in the subsequent analyses described below. TCC data for remaining trials were normalized to each subject's first trial with goggles by the following equation: *G*_*x*_ = (*T*_*x*_ / *T*_1_) × 100. *G* refers to the normalized time for a specific goggle trial number (*x*). *T*_1_ represents the TCC (in seconds) for the first trial with goggles on. The time metrics for *G* are expressed as a percentage of the time it took during the first trial with goggles on (*T*_1_). Thus, *G*_1_ is always equal to 100%.

Microsoft Excel (Microsoft Corp., Redmond, WA, USA) was used to determine the best fit adaptation curves to the normalized TCCs for each subject's nine trials of the FMT with goggles on (*G*_1_ – *G*_9_). The fit of four different types of functions were evaluated: exponential, logarithmic, polynomial, and power. Overall means of the R^2^ for the power function (mean *R*^2^ = 0.906) indicated it fit the data better than the exponential (mean *R*^2^ = 0.805), logarithmic (mean *R*^2^ = 0.878), and polynomial (mean *R*^2^ = 0.888) functions. Therefore, the power function was used to characterize the short-term, strategic adaptation rates for the nine FMT trials performed with goggles on. The power function used was *y* = *c*(*x*^α^), where *y* is the estimated normalized time, *c* is a constant, *x* is the goggle trial number (1–9), and α is an exponent value that can represent how steep the adaptation curve is. Specifically, the more negative values of α become, the steeper the curves are, indicating faster rates of strategic adaptation to the sensory discordant environment.

As the effect of SVS on normal strategic adaptation was paramount to this study, a criterion was established to exclude outliers displaying this lack of adaptation. It has been shown previously that not everyone displays typical strategic adaptation to certain tasks (Bock, 2005). Visual inspection of adaptation suggested that three subjects had extreme difficulty in improving their performance on the locomotor task with goggles, and therefore appeared to be statistical outliers, as the supplementary image shows (Appendix A). We then determined that these subjects' maximum improvement were at least two standard deviations below the mean improvement of all 27 subjects, and they were removed from further analysis. Thus, a total of 24 subjects were analyzed, which consisted of 12 subjects in each group (control, *n* = 12; stim, *n* = 12).

Statistical analysis for the data was performed using SPSS 20 (IBM Corp., Armonk, NY, USA). The specific variables assessed in the study were the normalized times from trials with goggles on (*G*_2_ – *G*_9_; *G*_1_ was not compared between the groups because all values would be equal to 100%), and the exponent values for the power trendlines indicating the adaptation rates (α) with goggles on. Shapiro-Wilk's tests and evaluations of Q-Q plots were utilized to check for data normality. Homogeneity of variance was assessed using Levene's tests for equality of variances with significance set at *p* < 0.05. One-tailed independent-samples *t*-tests were used to compare differences between the two groups (control vs. stim) with significance also set at *p* < 0.05.

## Results

**Figure 2:**
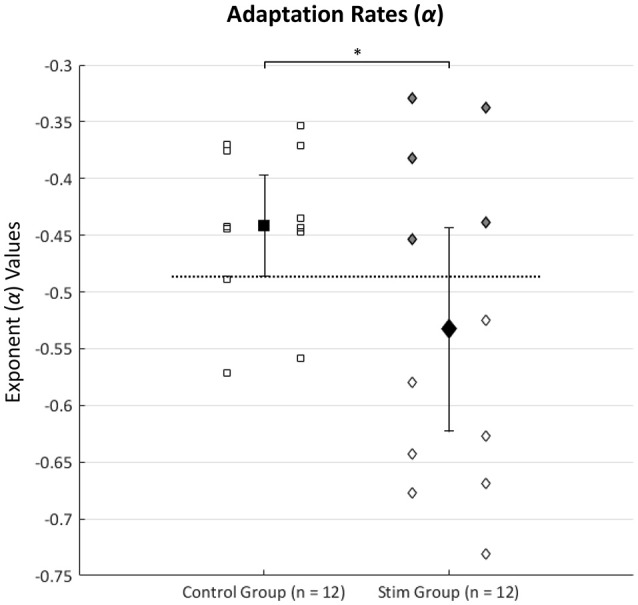
Shows each subject's exponent value (α) for the power equation [*y* = *c*(*x*^α^)] trendlines used to represent the strategic adaptation rates during the nine trials of the FMT done with goggles on. Values more negative indicate faster adaptation. Squares represent the control group while diamonds depict the stim group adaptation rates. Group means ± the 95% CI are represented by the larger dark filled shapes with error bars. The asterisk denotes significantly faster adaptation rates (^*^p < 0.05) in the stim group (*n* = 12) compared to the control group (*n* = 12). The top five gray shaded diamonds depict five subjects in the stim group whose adaptation rates were not faster than the lower bound of the control group 95% CI and were considered “non-responders” to SVS. Consequently, the bottom seven hollow diamond shapes from the stim group depict adaptation rates of those who were considered “responders” to SVS. The horizontal dotted line represents the cut-off criteria for “responders” and “non-responders” in the stim group (the lower bound of the control group's 95% CI, thus establishing the criteria that “responders” had to have faster adaptation rates than most of the control subjects).

Figure 2 shows each subject's exponent value (α) for the power function [*y* = *c(x*^α^*)*] used to represent the strategic adaptation rates during the nine trials of the FMT done with goggles on. The initial one-tailed independent-samples *t*-tests revealed significantly faster adaptation rates (α) in the stim (*n* = 12) group than the control (*n* = 12) group [*t*_(16.18)_ = 2.00, *p* = 0.031], but no significant differences were found between the two groups (control and stim) for any of the normalized TCC times completed during visual discordance. Multiple studies have noted that SVS does not always reveal an effect of improved balance performance in all subjects receiving the stimulation (Mulavara et al., 2011, 2015; Goel et al., 2015). We expected those individuals responding to SVS would display faster adaptation rates than most of the control subjects. Therefore, we identified a subgroup of “responders” as stim individuals whose adaptation rates (α) were faster (more negative) than the lower 95% confidence interval (CI) bound from the control group's mean adaptation rate. Having identified seven responders, we then performed a second analysis with independent-samples *t*-test comparisons between the control group (*n* = 12) and the “responders” (*n* = 7). Consequently, individuals in the stim group who did not have adaptation rates faster than the lower 95% CI bound of the control were defined as “non-responders”. Identification of the “responders” and “non-responders” subgroups within the stim group can also be indicated in Figure 2 by color shading.

**Figure 3:**
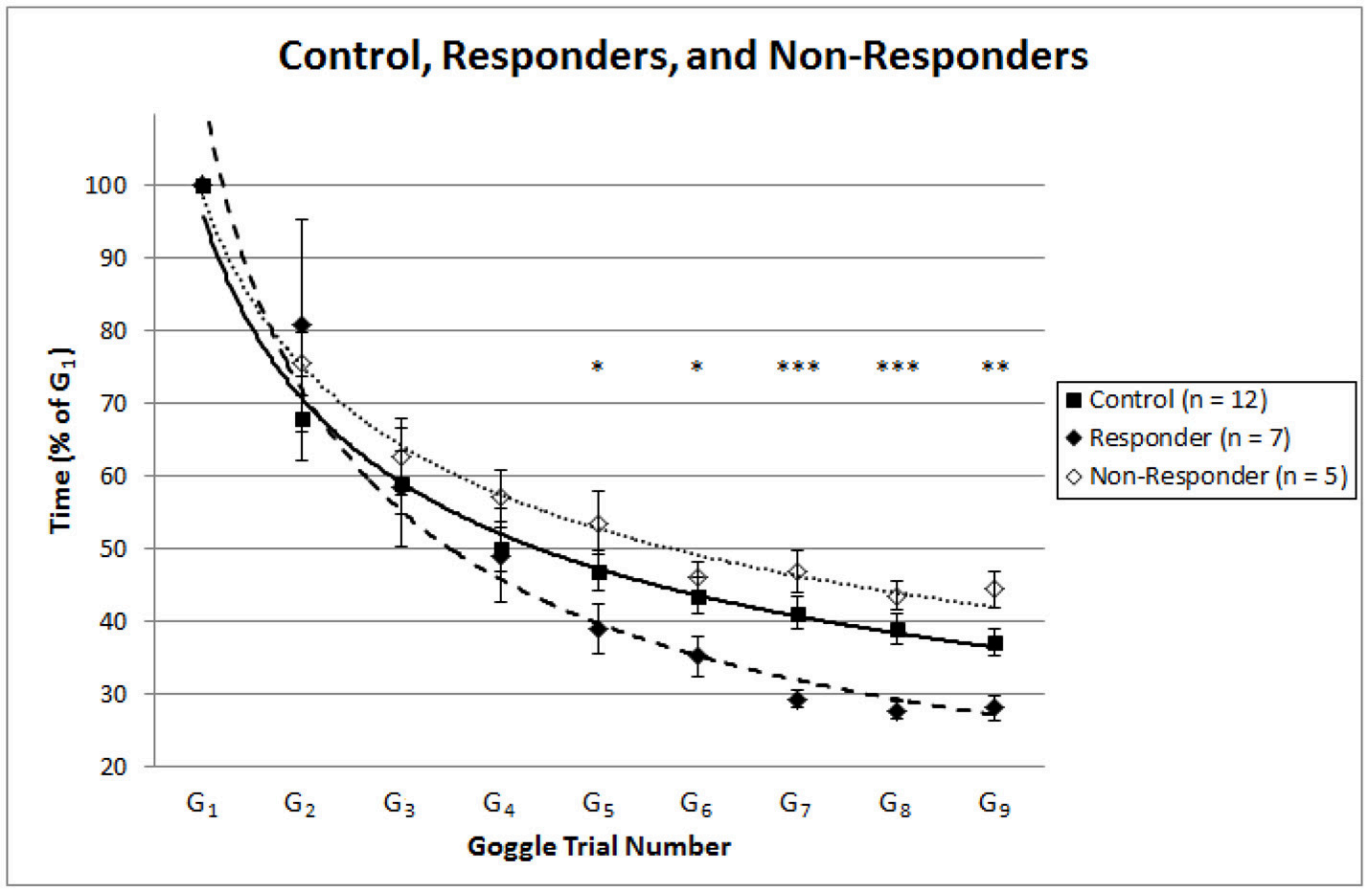
Depicts the normalized trial time means ± 1 standard error for the control group (dark squares), stim group “responders” (dark diamonds; *n* = 7), and stim group “non-responders” (hollow diamonds; *n* = 5). Power equation [*y* = *c*(*x*^α^)] trendlines were plotted from the trial time means to represent strategic adaptation curves for the control (solid line), “responder” (dashed line), and “non-responder” (dotted line) groups. Asterisks denote significantly faster trial times (^*^p < 0.05, ^**^p < 0.01, and ^***^p < 0.001) in the stim group “responders” (*n* = 7) compared to the control group (*n* = 12) for goggle trials five through nine (*G*_5_–*G*_9_).

In the second analysis when comparing the stim group “responders” (*n* = 7) to the control group (*n* = 12) during visual discordance, several significant comparisons emerged by the fifth trial. As Figure 3 indicates, normalized TCC for goggle trials five through nine (*G*_5_ – *G*_9_) were significantly faster with the “responders” of the stim treatment than the control group [*G*_5_: *t*_(17)_ = 1.78, *p* = 0.047; *G*_6_: *t*_(17)_ = 2.15, *p* = 0.023; *G*_7_: *t*_(16.11)_ = 4.64, *p* < 0.001; *G*_8_: *t*_(16.18)_ = 4.56, *p* < 0.001; *G*_9_: *t*_(17)_ = 3.27, *p* = 0.003]. As would be expected when we split the stim group by adaptation rates (α) falling above and below the lower bound of the 95% CI for the control group, the “responders” (*n* = 7) were confirmed to have significantly faster adaptation rates than the control (*n* = 12) group [*t*_(17)_ = 5.88, *p* < 0.001]. Thus, those responding to SVS had significantly faster adaptation rates than controls who received the zero stimulus, and these faster rates of adaptation led to significantly faster trial times by trial number five (*G*_5_) under conditions of visual discordance. Once reaching a significant level, these significantly faster trial times in responders continued to be maintained throughout the additional visual discordant trials performed in the experiment (*G*_6_ – *G*_9_), as Figure 3 shows. To assess whether there were differences in adaptation and normalized TCC between the control and our identified “non-responders”, we conducted two-tailed independent-samples *t*-tests. These results revealed no difference in adaptation rate, and at only a single time point (*G*_9_) were the “non-responders” slower than the control group.

Rated sensations for degree of difficulty balancing were generally larger for the goggle trails compared to the baseline conditions (*B*_1_ – *B*_3_), with *G*_1_ being the greatest reported average (*G*_1_ mean rating = 4.1). These higher ratings indicated that the vision reversing prisms posed a fairly difficult challenge to subjects' balance during locomotion. Perceived nausea and electrode irritation ratings were very low on average (≤ 1.1 for each sensation) and did not change much throughout the trials (mean change ≤ 0.3 units for each sensation).

## Discussion

In this study, we investigated if SVS could improve locomotor performance within an adaptation paradigm. We hypothesized that subthreshold levels of electrical broadband white noise delivered to the vestibular system could improve adaptation to a novel sensory discordant environment. The data suggests that adaptation rates were faster in the subjects who received SVS than the controls. Moreover, in a subgroup of subjects who were responsive to SVS, short-term strategic adaptation to the visual and somatosensory discordant environment of the FMT seemed improved relative to controls and non-responders by the fifth trial. We proposed the improvements in short-term strategic adaptation seen in those responsive may have been caused by better detection of vestibular input provided via the SR phenomenon.

The exact mechanism by which SR occurs requires further elucidation. It has been proposed that Galvanic vestibular stimulation acts on spike trigger zones of vestibular afferents (Goldberg et al., 1982, 1984). However, other studies have shown that Type I mammalian vestibular hair cells may have mechanical responses evoked by low frequency electrical current, where rotational mechanical characteristics of the stereocilia may be changed, modifying the hair bundle position, and effecting transduction during head tilt and acceleration (Zenner and Zimmermann, 1991; Zenner et al., 1992). Additionally, it has been reported that subthreshold SVS may improve postural control by facilitating the vestibulo-spinal control system or other non-dopaminergic pathways as has been suggested in Parkinson's disease patients as well as healthy individuals (Pal et al., 2009; Mulavara et al., 2011; Samoudi et al., 2015). All of the above mechanisms may play an important role in the improved behavioral responses observed with SVS. Regardless of the exact mechanism responsible for the SR phenomenon, the improvements to locomotion and postural control when applying subthreshold amounts of a bipolar electrical broadband white noise stimulus to the vestibular system have been well documented (Mulavara et al., 2011, 2015; Goel et al., 2015; Samoudi et al., 2015). Studies have suggested that SVS is capable of improving postural control and locomotor stability in numerous populations with balance deficits, individuals with neuropathies, the elderly, recurrent fallers, and those with Parkinson's disease (Mulavara et al., 2011, 2015; Goel et al., 2015; Samoudi et al., 2015). Furthermore, studies observing these benefits of SVS on balance performance have done so with only a few minor or no adverse effects reported in subjects (Goel et al., 2015; Samoudi et al., 2015). Consistent with this prior research, our subjects did not report any significant adverse effects credited directly to the SVS. The most commonly reported sensations were slight nausea or vertigo, minor headache from the head strap, and difficulty balancing attributed to the sensory discordant conditions with up/down vision reversing prism googles on, experienced during the FMT.

It is worth noting that not all subjects receiving the SVS responded in a manner showing improved strategic adaptation performance beyond that of the controls. Figure 2 shows that five out of the 12 subjects receiving vestibular stimulation did not have adaptation rates (α) better than the lower bound of the 95% CI from the control mean. We considered these subjects to be “non-responders” to the vestibular stimulation they received. In our previous studies exploring SVS, we have noted rates of “non-responders” to be around 30% (Mulavara et al., 2011, 2015; Goel et al., 2015; Samoudi et al., 2015). The results in the present study suggests a slightly higher rate of non-responders (5/12 = 41.7%).

The present results suggest that application of low amplitude SVS may be able to assist a countermeasure that has been proposed to improve locomotor capabilities in astronauts after spaceflight. This countermeasure utilizes the phenomenon of adaptive generalization to train the sensorimotor adaptability (SA) capabilities of astronauts (Bloomberg et al., 2015). Adaptive generalization suggests that repeatedly adapting to conflicting sensory environments fosters people's ability to adapt to new, novel displacements (Welch et al., 1993). Practice solving certain classes of motor control problems enables them to adapt faster or “learn to learn.” The type of training to make use of this concept of adaptive generalization has been termed SA training. It has been suggested that SA training programs that expose astronauts to varying sensory input and balance challenges can enhance their ability to assemble appropriate motor patterns in sensory discordant environments, thus improving their ability to quickly adapt. In addition to the lack of postural control and locomotor capabilities post-flight in astronauts which have been well documented (Paloski et al., 1992, 1994; Paloski, 2000; Mulavara et al., 2010), an increased reliance on visual feedback during recovery from spaceflight has also been reported (Reschke et al., 1998). Numerous studies have reported that subjects relying more on vision during locomotion have difficulty adapting walking and postural control strategies when in novel sensorimotor discordant environments (Hodgson et al., 2010; Brady et al., 2012; Eikema et al., 2013). It is suggested however that subjects who are more visually dependent can be trained to better utilize other sensory modalities such as those provided by vestibular and somatosensory inputs (Wood et al., 2011; Mulavara et al., 2015). Future studies in SR should address the issue of sensory bias and evaluate whether or not improved vestibular and somatosensory signal detection can reduce reliance on vision in postural control and locomotion tasks. If those who are more visually dependent can be found to explore other sensory modalities better with the assistance of SR, then effectiveness of SA training may be further enhanced.

As noted, not all subjects responded to SVS, at least not in a measurable way. Reasons for unresponsiveness to the noise stimulus can incur much speculation. Perhaps receiving noise in these individuals at amplitudes of half their perceptual thresholds was not ideal, although we have previously shown ideal amount of SVS provided to improve postural control in a Romberg posture task to be around 46 to 53% of perceptual thresholds (Goel et al., 2015). A recent study found average optimal improvement in locomotor stability to be achieved at vestibular stimulus levels approximately 35% of the perceptual threshold (Mulavara et al., 2015), thus optimal dynamic stability might be achieved at slightly less levels of stimulation. Additionally, the current study did not take into account the potential for various levels of internal noise to exist between subjects. It has been hypothesized that behavioral responses may be optimized by the interaction between the external noise applied and the internal noise already present within the CNS, such that with high levels of internal noise present, less external noise may be needed for optimal performance and vice versa (Aihara et al., 2008; Goel et al., 2015). Adjusting levels of vestibular stimulation received by a metric of internal noise could potentially increase effects of SVS seen on FMT performance. Future studies need to focus on ways to identify these potentially high internal noise individuals that may be unresponsive to the addition of external noise prior to testing, or at least find other objective measures other than task performance which can help to identify potential “responders” and “non-responders” to the noise stimulation. It is also possible that using healthy and relatively young individuals in this study provided us with some subjects whose vestibular systems were already performing at near optimal levels, and thus gaining more sensory input through the additional noise provided may not have been viable (Priplata et al., 2003). Subjects with prior impaired balance capabilities may have shown a greater effect of SVS, had they been used in the present study. Effects of SR on locomotor performance have previously been reported to occur on somewhat of a continuum, where those individuals who display greater gait variability seem to benefit more from SR than younger subjects who show less variability (Galica et al., 2009). In other words, those with greater decrements in their gait ability seem to have a greater chance for SR to improve their performance. Thus, understanding and identifying the limitations of the SR approach via vestibular stimulation is important from a variety of standpoints, relating to the efficacy of using it appropriately in various rehabilitation countermeasures to make it more personalized based on individual characteristics (Seidler et al., 2015; Seidler and Carson, 2017).

A few limitations should be of note for this study. First, the sample size is relatively small. The few number of subjects per group in the analysis could have made it difficult to find significant differences between some comparisons, as well as overinflate the importance of individuals' data when making comparisons that divided them into even smaller groups. As using an adaptation paradigm was paramount to the study though, it was not possible to make within subject comparisons and have subjects serve as their own control. Thus, the total number of subjects collected had to be divided into the between subject comparison groups. Limited funds and time did not make collection of more subjects a viable option. It is suggested that future research observing effects of SVS on adaptation should be conducted with a greater number of participants. Additionally, although the subjects in this study were blinded as to whether or not they were receiving zero or subthreshold SVS, it was not possible to conduct this study in a double-blind fashion. The researcher who assigned the stimulus profile to the current stimulator needed to be present to ensure the device was working correctly and assist in safely spotting the subjects. Great care however, was taken to ensure that the exact same instructions were given to every subject before each trial, and spotting techniques did not change between trials or subjects. Finally, the sole performance metric used in this study was TCC. Although it appeared to be a valid metric to assess postural control during the FMT, as individuals with more difficulty balancing were slower and it has been utilized previously (Reschke et al., 2009; Mulavara et al., 2010), future studies should proceed to collect other forms of kinematic and kinetic data during similar adaptation tasks to more completely characterize the postural control.

## Conclusion

Our study indicated that short-term locomotor adaptation to a somatosensory and visually discordant environment may be improved, in some individuals when adding subthreshold amounts of broadband binaural bipolar stochastic electrical stimulation to the vestibular system. The acceleration of adaptation as displayed in improved performance is believed possible as a result of SR occurring within the vestibular system. Such improvements in locomotor adaptation could have implications for use as a countermeasure or perhaps in enhancing other countermeasures like SA training programs that strive toward optimizing adaptation capabilities of astronauts who incur sensorimotor challenges during gravitational transition periods (Goel et al., 2015). Results also suggest that SVS may be applicable for improving locomotor performance in populations with balance deficits, such as those with Parkinson's disease (Samoudi et al., 2012, 2015), stroke, diabetic neuropathy, recurrent fallers, or the elderly (Mulavara et al., 2011, 2015). It is suggested that future directions of research in this area should also explore more the possible explanations as to why some subjects appear more responsive to SVS than others, such as observing relationships in sensitivities of various sensory channels (e.g., somatosensory or proprioception, vestibular, or visual). Potential carry-over effects of SVS improving balance should also be studied. Kim noted effects of noisy SVS on brain rhythms were present several seconds after stimulation ceased (Kim et al., 2013), and thus the potential for sustained balance improvements after stimulation warrants further investigation.

## Author contributions

DT helped with study design, collected data, analyzed the data, and wrote the manuscript. YD helped collect the data and edit the manuscript. CL helped with study design and editing the manuscript. JB helped with study design and editing the manuscript. AM helped with study design, analyzing the data, and editing the manuscript.

### Conflict of interest statement

The authors declare that the research was conducted in the absence of any commercial or financial relationships that could be construed as a potential conflict of interest.
