# Supplementary material for: Efficacy of Stochastic Vestibular Stimulation to Improve Locomotor Performance During Adaptation to Visuomotor and Somatosensory Distortion
Source: Front Physiol. 2018 Mar 29;9:301. doi: 10.3389/fphys.2018.00301 (PMC5885191; doi:10.3389/fphys.2018.00301)

Appendix A: This figure graphs the normalized TCC of the three excluded outliers as well as the mean ±1 SD of all other 24 subjects tested during the goggle trials ($G_1 – G_9$). The values of G_2, G_3, and G_4 are not included in this chart for outlier 2, as those three trials for that subject were slower than their initial G_1 trial (> 100% of G_1.).

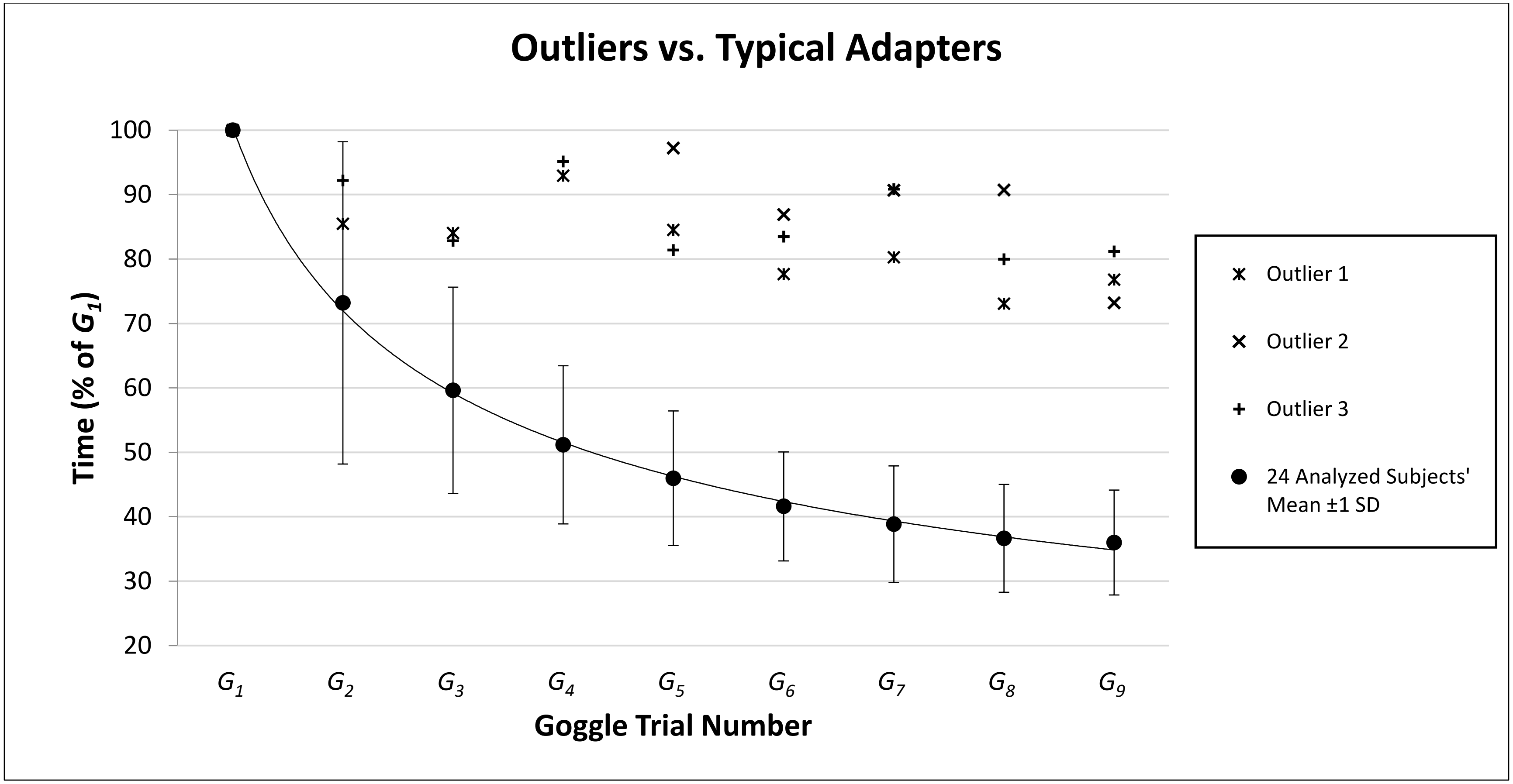

Supplement: Supplementary file 1 [file Image1.PDF]
